# In-utero exposure to phenols and phthalates and the intelligence quotient of boys at 5 years

**DOI:** 10.1186/s12940-018-0359-0

**Published:** 2018-02-20

**Authors:** Dorothy Nakiwala, Hugo Peyre, Barbara Heude, Jonathan Y. Bernard, Rémi Béranger, Rémy Slama, Claire Philippat, The EDEN mother-child study group, M. A. Charles, M. de Agostini, A. Forhan, B. Heude, P. Ducimetière, M. Kaminski, M. J. Saurel-Cubizolles, P. Dargent-Molina, X. Fritel, B. Larroque, N. Lelong, L. Marchand, C. Nabet, I. Annesi-Maesano, R. Slama, V. Goua, G. Magnin, R. Hankard, O. Thiebaugeorges, M. Schweitzer, B. Foliguet, N. Job-Spira, JY. Bernard, B. de Lauzon-Guillain, A. Germa, F. Pierre, J. Lepeule, J. Botton

**Affiliations:** 1 Institute for Advanced Biosciences (IAB), INSERM U1209, CNRS UMR 5309, Université Grenoble Alpes, 38000 Grenoble, France; 2 Assistance Publique-Hôpitaux de Paris, Robert Debré Hospital, Child and Adolescent Psychiatry Department, Paris, France; 3 Cognitive Sciences and Psycholinguistic Laboratory, Ecole Normale Supérieure, Paris, France; 4 U1153 Epidemiology and Biostatistics Sorbonne Paris Cité Research Centre (CRESS), Early Origin of the Child’s Health and Development (ORCHAD) Team, Inserm, Villejuif, France; 5 Université Paris Descartes, Villejuif, France; 6 Singapore Institute for Clinical Sciences (SICS), Agency for Science, Technology and Research (A*STAR), Singapore, Singapore; 7 Inserm U1085–IRSET, Université Rennes 1, Rennes, France

**Keywords:** Cognitive function, Endocrine disruptors, Phthalate, Intelligence quotient, Bisphenol A, Parabens, Triclosan, Prenatal exposure

## Abstract

**Background:**

There are concerns that developmental exposure to endocrine disrupting chemicals such as phenolic compounds and phthalates could affect child cognitive function. Epidemiological studies tackling this question have mainly focused on phthalate metabolites and bisphenol A, but not on the other phenolic compounds. Our study aimed to assess the relationship between in-utero exposure to phthalates, bisphenol A and other phenolic compounds (parabens, triclosan, dichlorophenols and benzophenone-3) and the Intelligence Quotient (IQ) of boys at 5–6 years.

**Methods:**

In 452 mother-son dyads from the French EDEN cohort, we measured 11 phthalate metabolites and 9 phenolic compounds (4 parabens, benzophenone-3, bisphenol A, 2 dichlorophenols and triclosan) in spot urine samples collected between 22 and 29 gestational weeks. Verbal and performance IQ of children were assessed at 5–6 years by a psychologist using the Wechsler Preschool and Primary Scale of Intelligence (WPPSI). We used adjusted Structural Equation Models (SEM) combined with Benjamini and Hochberg false discovery rate correction to assess the associations between maternal urine phenol and phthalate metabolite concentrations considered simultaneously and the boys’ IQ.

**Results:**

No phenol or phthalate metabolite concentration was negatively associated with the boys’ verbal or performance IQ (uncorrected *p*-values ≥0.09). Mono(3-carboxypropyl) phthalate tended to be associated with increased verbal IQ (*β* = 0.136, 95% confidence interval, 0.01; 0.27). This association disappeared after correction for multiple comparison (corrected *p*-value, 0.71).

**Conclusion:**

Our results did not provide evidence of an inverse association between in-utero exposure to phenols or phthalates and verbal and performance IQ among boys. Since phenols and phthalates may have sex-specific effects, these null findings cannot be generalized to girls. Limitations included use of a single spot urine sample to assess exposures and lack of consideration of postnatal exposures.

**Electronic supplementary material:**

The online version of this article (10.1186/s12940-018-0359-0) contains supplementary material, which is available to authorized users.

## Background

Phenols and phthalates are synthetic compounds with wide application including, manufacture of polycarbonate plastics (bisphenol A), UV-filters in sun screens (benzophenone-3), microbicides in toothpaste and soaps (triclosan), preservatives in food, cosmetics and personal care products (parabens), plasticizers in food packing material, personal care products, medications and residential floor materials (phthalates). Exposure to phenols and phthalates is ubiquitous in the general population and these compounds have been detected in urine samples of mothers and their children [1], amniotic fluid [2, 3] and umbilical cord blood [4]. Some phenols and phthalates have endocrine disrupting capabilities [5–7] and can indeed interfere with normal synthesis, metabolism, secretion, transport, receptor binding, action, or elimination of endogenous hormones, including the thyroid and sex hormones [8–11] that are involved in fetal brain development [12, 13]. Disruption of the normal process of brain development can lead to impaired or abnormal development of cognitive skills [14]. Functionally, cognitive development can be classified in several interrelated domains such as intelligence, attention, language and communication, executive function, visuospatial abilities and learning and memory [15]. In this study, we assessed effects on the intelligence quotient (IQ), a psychometric approach that assesses the child’s ability to complete verbal and performance tasks.

Several publications have explored the associations between prenatal exposure to phenols and behavior [6, 16], a social-emotional domain that encompasses developmental adaptivity of a child to its social environment and not under the scope of this paper. Only five studies, majorly focusing on bisphenol A, have examined child cognitive development in relation to urinary [17–20] and umbilical cord blood [21] bisphenol A concentrations. Among these, three assessed child IQ and neither found any association with maternal urinary bisphenol A concentrations except one suggesting a negative association with IQ and bisphenol A measured in cord blood [21]. To our knowledge, no study has examined IQ in relation to prenatal exposure to other phenols such as triclosan, dichlorophenols, parabens or benzophenone-3.

Regarding phthalates, epidemiological studies on the cognitive function mainly assessed associations with the mental development index of the Bayley Scales of Infant Development (BSID) or with IQ of children between 6 months and 11 years of age. With two exceptions reporting null associations in relation to IQ [22, 23], most of these studies reported negative associations between maternal urinary concentrations of several phthalate metabolites with the mental development index at 6 months [24], 2 years [25, 26] and 3 years [27] and full scale IQ of children at 7 [28]. Depending on the study, some of the phthalates incriminated were mono-n-butyl phthalate (MBP) [24, 26, 28], di(2-ethylhexyl) phthalate (DEHP) metabolites [24, 26], mono-isobutyl phthalate (MIBP) [28], monobenzyl phthalate (MBZP) [28] and mono(3-carboxypropyl) phthalate (MCPP) [25].

In summary, previous human literature did not report clear association between prenatal exposure to bisphenol A and child IQ, while negative associations have been reported by most studies for phthalates, at various ages. We were not able to identify epidemiological studies that explored associations between phenols other than bisphenol A and child cognition.

In this study, we examined associations between maternal urinary phenol and phthalate metabolite concentrations and IQ of boys at 5–6 years with the aim of replicating deleterious effects between phthalate and the child cognitive function observed in previous studies [24–28] and providing exploratory results regarding the potential effects of prenatal exposure to phenols such as triclosan and parabens on child cognitive function. With regards to previous literature in humans that did not report clear association between prenatal exposure to bisphenol A and cognitive function [17–20], we were not expecting an association with child IQ for this chemical.

## Methods

### Study population

The study population was a sample of mother-son pairs of the EDEN (*Etude des Déterminants pré et post natals du développement et de la santé de l’Enfant*) cohort [29]. The EDEN cohort consists of 2002 pregnant women recruited before the end of the 28th gestational week from February 2003 through January 2006 in the obstetric departments of Nancy and Poitiers university hospitals, France. The cohort received approval from the ethics committee of Kremlin-Bicêtre University hospital. To be included, participants gave informed written consent for themselves and for their children.

Among the 998 boys of the EDEN cohort born alive, 452 had IQ assessments at 5 years and urinary concentrations of phenols and phthalate metabolites assessed in maternal urine. Urinary concentrations of phenols and phthalate metabolites were assessed in a framework of previous projects that evaluated associations between these compounds, male genital malformation [30] and growth [31–33], explaining why we focused on boys. Inclusion criteria for biomarker assessments was being a boy, having at least one urine sample available during pregnancy and having data on growth during the pre and postnatal period (up to 3 years). While this prevents generalizability of our findings to females, focusing on one sex is not a source of bias. Moreover, in the context where sex specific effects are plausible [17, 24, 25, 27], a study restricted to one sex will indeed have higher statistical power than a study of similar sample size including both sexes in which two sex-specific analyses are conducted.

### Assessment of IQ

IQ was assessed at an average age of 5.7 (Standard Deviation (SD): 0.1) years using the French version of the Wechsler Preschool and Primary Scale of Intelligence-Third Edition (WPPSI-III) [34]. This scale was translated, culturally adapted to French norms, and calibrated in a sample of 999 children representative of the French children between aged 2.5 and 7.25 years by the ECPA (Editions of the Centre in Applied Psychology) [35]. Assessments were carried out by two trained psychologists using the seven core subtests to compute the verbal and performance IQ scores. The seven subtests include: information (number of correct answers to questions that address a broad range of general knowledge topics, 34 items), vocabulary (number of words correctly defined, 25 items), word reasoning (number of concepts correctly identified from a series of clues, 28 items), block design (number of correct designs recreated using blocks, 20 items), matrix reasoning (number of matrices correctly completed, 29 items), picture concepts subtest (number of correct selections of 2 or 3 pictures with common characteristics, 28 items) and coding (number of correct symbols copied corresponding to geometric shapes, 59 items) [36].

### Exposure assessment

We measured concentrations of bisphenol A, benzophenone-3, triclosan, 2,4 and 2,5 dichlorophenol, methyl-, ethyl-, propyl- and butyl-paraben along with 11 phthalate metabolites from maternal urine samples collected during pregnancy between 22 and 29 gestational weeks using online solid phase extraction-high performance liquid chromatography-tandem mass spectrometry [37, 38]. Creatinine, a proxy of urine dilution, was also measured. Assessments were performed at the National Center for Environmental Health laboratory at the CDC in Atlanta, Georgia, USA.

For concentrations below the limit of detection we used the instrumental reading values. To allow ln-transformation, instrumental reading values equal to 0 (i.e. indicative of no signal) were replaced by the lowest instrumental reading value divided by the square root of two [32]. To limit the impact of between subject variations in urine sampling conditions, concentrations were standardized for collection conditions such as hour of sampling, gestational age at collection, duration of storage at room temperature before freezing, day of sampling, year of biomarker assessments at CDC (2008 or 2011) and creatinine levels. This was done using a 2 steps statistical approach [32, 39]. We first studied the influence of each sampling condition and creatinine levels on the measured biomarker concentrations using adjusted linear regression. We then predicted standardized concentrations using the effect estimates for each sampling condition that was associated with the biomarker concentrations (*p*-value < 0.2) and the measured biomarker concentrations [39]. The standardized concentrations assumed that all samples were collected under similar conditions and were used in all of our analyses.

### Confounding

Confounders were identified a priori from literature and included variables associated with both exposure and the outcome, without being likely consequences of the exposures thereof, associated with the outcomes only. These included center of recruitment (Poitiers/Nancy), maternal age (continuous), parity (nulliparous/≥1 child), maternal body mass index (BMI, continuous), parental education level (average number of years spent in school by mother and father), breastfeeding duration (never/breastfed), monthly household income (≤1500, 1500 – 3000 and ≥3000 Euros), smoking during pregnancy (yes/no), maternal psychological difficulties during pregnancy (yes/no), child cognitive stimulation (continuous) and finally, child age at assessment (continuous). In our main analysis, we used crude IQ subscale scores and adjusted for child age because we observed negative associations (results not shown) between the age standardized scores with child age at IQ assessment. Child cognitive stimulation was assessed using items from the short form of the Home Observation for the Measurement of the Environment Scale questionnaire (HOME) at 5 years. The maternal psychological difficulties score during pregnancy was constructed by combining scores of the Center for Epidemiologic Studies Depression Scale Revised (CESD) and of the State-Trait Anxiety Inventory (STAI) two questionnaires designed to assess depression and anxiety respectively. Among the 452 mother-son pairs included in the study, several covariates had missing values. We used multiple imputation using the *mi impute chained* command in STATA14 with 5 imputed datasets [40] to handle the missing data for the HOME score (*N* = 25, 5.5%), BMI (*N* = 4, 0.9%), parity, income and maternal depression and psychological difficulties (*N* = 1, 0.2%).

### Association between biomarker concentrations and the boys’ IQ

**Fig. 1:**
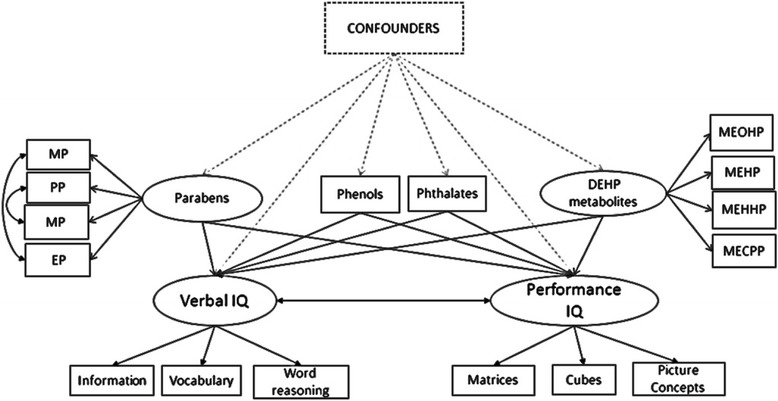
Path diagram illustrating associations between phenols, phthalate metabolites and IQ of boys. Shapes representations: circles: latent variables, rectangles: observed variables, dashed and solid arrows: linear relationships, double headed arrows: correlations. Abbreviations: MP: methyl paraben, EP: ethyl paraben, PP: propyl paraben, BP: butyl paraben, DEHP: di(2-ethylhexyl) phthalate metabolites, MECPP: mono(2-ethyl-5-carboxypentyl) phthalate, MEHHP: mono(2-ethyl-5-hydroxyhexyl) phthalate, MEOHP: mono(2-ethyl-5-oxohexyl) phthalate, MEHP: mono(2-ethylhexyl) phthalate. Phenols represents 2,5-dichlorophenol, benzophenone 3, bisphenol A and triclosan. Phthalates represents mono-n-butyl phthalate, monobenzyl phthalate, monocarboxy-isononyl phthalate, monocarboxy-isooctyl phthalate, mono(3-carboxypropyl) phthalate, monoethyl phthalate and mono-isobutyl phthalate. Confounders include maternal age, age of child at IQ assessment, HOME stimulation score, maternal body mass index before pregnancy, parental education level, parity, monthly revenue, breast feeding duration and maternal psychological difficulties during pregnancy

All biomarker concentrations were ln-transformed before analysis. We used Structural Equation Models (SEM) to study the associations between the urinary biomarker concentrations and the boys’ IQ scores. SEM simultaneously assesses relationships between one or more independent variables and one or more dependent variables. They comprise of two parts, 1) a measurement model based on factor analysis that relates observed variables to latent constructs and 2) a structural model, based on ordinary regression which describes the relationships between the generated latent exposure and outcome constructs. Latent constructs underlying the IQ outcomes included 2 factors: the verbal and performance factors, that were determined a priori from previous literature [41, 42]. The Confirmatory Factor Analysis we performed on our data also supported this two-factor structure (Additional file 1: Table S1). The performance IQ factor was constructed using the matrix reasoning, block design and picture concept scores while verbal IQ factor was constructed using the information, vocabulary and word reasoning scores of the WPPSI (Fig. 1). For the exposures, we first considered 3 latent variables based on factorial analysis: one predicted by the 2 dichlorophenols, one by the 4 parabens and one by the 4 DEHP metabolites. Because the latent variable based on the two dichlorophenols caused the model to be unidentified, we decided not to include a latent variable for the dichlorophenols and instead included only one of the dichlorophenols (2,5 dichlorophenol) as an observed variable. The final model had 2 latent exposure variables representing paraben and DEHP exposures and all other biomarker concentrations were added as observed variables (Fig. 1). We allowed the error covariance between, methyl and propyl paraben, butyl and ethyl paraben and the latent variable of parabens with monoethyl phthalate to be freely estimated. Estimating covariance between the DEHP metabolites freely did not improve the fit of the model and was not included in our final model. We reported standardized association estimates, expressed as the change in SD of IQ scores associated with a 1-SD increase in the ln-transformed biomarker concentrations.

We tested 26 associations (2 outcomes * 13 biomarkers) and cannot exclude that part of the associations observed were due to chance. For this reason, in addition to the regular *p*-value we presented in the results section the *p*-values corrected for multiple testing using the Benjamini and Hochberg false discovery rate (FDR) method [43].

#### Sensitivity analysis

In the sensitivity analysis, we ran a SEM model using only one construct for IQ (full scale IQ) constructed using all seven subscales of the WPPSI-III (Additional file 1: Figure S1) We also performed analysis using only complete cases (no imputation of the missing covariates). Because exposure levels and IQ scores differed by recruitment centers, we performed additional analysis stratified by center. While commonly used in psychology and sociology, SEM have so far had limited use in environmental epidemiology [44, 45]. For this reason, we also conducted more classic analyses using the manual based IQ scores instead of latent constructs and run linear regression models (one model per outcome and exposure). We explored the shape of the associations between phenols, phthalates and IQ by studying exposures categorized as tertiles.

Analysis was carried out using MPLUS 6 (Muthen & Muthen, Los Angeles, CA, USA) and STATA/SE 14 (StataCorp, College Station, TX, USA) statistical softwares.

## Results

### Population characteristics

The majority of mothers in the study population (63%) were recruited from Poitiers. On average, mothers were 29.9 (SD: 4.7) years old and 46% were nulliparous. Fifty-five percent of the mothers and 44% of the fathers had more than 2 years of education after high school. Twenty-eight percent of the mothers reported no breast-feeding at all and about 23% smoked during pregnancy (Table 1). Compared to mother son-pairs of the EDEN cohort not included in our analysis, pairs in our study population were more likely to be from Poitiers, from households that earned > 1500 euros/month, and less likely to have smoked during pregnancy (Table 1). Associations between maternal characteristics and child IQ are shown in Additional file 1: Table S2.

**Table 1:** Characteristics of the study population and the mother-son pairs of the French EDEN cohort

	Study population (*N* = 452)^a^		Mother-son pairs not in the study population (*N* = 546)^c^		*P*values^d^
Characteristics	*N*	(%)	*N*	(%)	
Recruitment center					
Poitiers	285	(63)	248	(45)	
Nancy	167	(37)	298	(56)	< 0.001
Parity					
Nulliparous	208	(46)	227	(41)	
≥ 1	243	(53)	319	(58)	
Missing	1	(0.2)			0.15
Maternal education					
≤ 2 years after high school	197	(44)	262	(48)	
High school + 2 years	105	(23)	114	(21)	
High school + 3 years	145	(32)	152	(28)	
Missing	5	(1)	18	(3)	0.22
Paternal education					
≤ 2 years after high school	224	(50)	259	(47)	
High school + 2 years	97	(21)	91	(17)	
High school +3 years	104	(23)	112	(21)	
Missing	27	(6)	84	(15)	0.48
Household monthly revenue (euros)					
≤ 1500	63	(14)	107	(17)	
1500–3000	275	(61)	296	(57)	
≥ 3000	113	(25)	135	(25)	
Missing	1	(0.2)	8	(1)	0.04
Smoking during pregnancy					
No	348	(77)	383	(70)	
Yes	102	(23)	161	(29)	
Missing	2	(0.4)	2	(1)	0.01
Maternal psychological difficulties^b^					
No	321	(71)	356	(65)	
Yes	130	(29)	186	(34)	
Missing	1	(0.2)	4	(1)	0.06
Was breastfeed					
Never	127	(28)	143	(26)	
Yes	325	(72)	396	(72)	
Missing			7	(1)	0.58
	Mean	(SD)	Mean	(SD)	
Maternal age at pregnancy	29.9	(4.7)	28.7	(5.0)	< 0.001
Total HOME score	17.3	(2.3)	17.0	(2.2)	0.14
Body Mass Index (kg/m^2^)	23.5	(4.6)	22.9	(4.5)	0.05
Child age at IQ assessment	5.7	(0.1)	5.7	(0.2)^c^	0.73

*Abbreviations*: *Home* Home Observation Measurement of the Environment, *IQ* Intelligence Quotient

^a^Includes mother-child pairs with biomarker assessments and IQ scores at 5–6 years

^b^Maternal depression or anxiety during pregnancy assessing using the CESD and the STAI respectively

^c^Includes boys for whom IQ was assessed *N* = 546

^d^*p*-value for comparison between subjects with complete data for IQ and biomarker assessments and those missing either data or both generated using independent X^2^ and t-tests

Most of the compounds assessed were detected in more than 93% of the urine samples except for triclosan, ethyl and butyl paraben that were detected in 73 to 84% of the samples (Table 2). As expected, correlations between the 4 parabens (spearman’s coefficients; 0.46 to 0.82), the 2 dichlorophenols (spearman’s coefficients: 0.69) and the DEHP metabolites (spearman’s coefficients; 0.78 to 0.98) were high. The other coefficients of correlation were in the − 0.11 to 0.61 range (Additional file 1: Table S3).

**Table 2:** Concentrations^a^ of phenols and phthalate metabolites in maternal urine (*N* = 452^b^ mother-son pairs of the EDEN mother child cohort)

Compounds (μg/L)	LOD (μg/L)	% < LOD	Percentiles		
			5th	50th	95th
Phenols					
2,4-dichlorophenol	0.2	2	0.3	1.0	10.0
2,5-dichlorophenol	0.2	0	1.7	9.2	305
Bisphenol A	0.4	0.4	0.9	2.4	9.3
Benzophenone-3	0.4	7	<LOD	2.2	63.7
Triclosan	2.3	18	<LOD	25.6	691
Methyl paraben	1	0	8.0	101	1529
Ethyl paraben	1	27	<LOD	3.4	689
Propyl paraben	0.2	1	0.5	13.4	255
Butyl paraben	0.2	14	<LOD	2	57.7
Phthalates					
Monoethyl phthalate (MEP)	0.6	0	21.9	100	591
Mono-n-butyl phthalate (MBP)	0.2	0	11.6	44.6	444
Mono-isobutyl phthalate (MiBP)	0.2	0	11.8	38.8	168
Mono(2-ethyl-5-carboxypentyl) phthalate (MECPP)	0.2	0	12.5	39.2	176
Mono(2-ethyl-5-hydroxyhexyl) phthalate (MEHHP)	0.2	0	6.9	29.0	106
Mono(2-ethyl-5-oxohexyl) phthalate (MEOHP)	0.2	0	5.8	23.3	87
Mono(2-ethylhexyl) phthalate (MEHP)	0.5	3	1.5	7.6	37
Monobenzyl phthalate (MBzP)	0.3	0	4.7	18.9	114
Monocarboxy-isooctyl phthalate (MCOP)	0.2	0	1.1	4.0	19
Mono(3-carboxypropyl) phthalate (MCPP)	0.2	0	0.8	2.0	9.4
Monocarboxy-isononyl phthalate (MCNP)	0.2	1	0.4	1.3	9.7
Creatinine			0.4	1.0	1.8

^a^Concentrations were standardized for creatinine concentrations and sampling conditions including hour of sampling, gestational age at collection and duration of storage at room temperature before freezing, day of sampling, year of biomarkers assessment

^b^Restricted to mother-son pairs with biomarker concentrations and IQ scores at 5–6 years available

### Associations between biomarker concentrations and boys’ IQ scores

After adjustment and imputation of the missing covariates, SEM with two latent constructs underlying the IQ outcomes converged normally, and fit indices suggested a statistically good fit: root Mean Square Error of Approximation (RMSEA) was 0.048, Comparative Fit Index (CFI) was 0.930, Tucker-Lewis fit index (TLI) was 0.921. The standardized factor loadings for indicators of both outcomes and exposure latent factors were uniformly high (standardized factor loadings > 0.53) and significant (*p*-values < 0.001), suggesting convergent validity [46] (Additional file 1: Table S1).

The only significant (uncorrected *p*-value below 0.05) association we observed was a positive association between MCPP and verbal IQ (*β* = 0.136 SD of IQ scores for an 1-SD increase in the ln-transformed MCPP concentration; 95% CI: 0.006; 0.266, Table 3). This association did not remain significant after correction for multiple comparison (corrected *p*-value = 0.71). No other phenol or phthalate was associated with performance or verbal IQ (uncorrected *p*-values ≥0.09, Table 3).

**Table 3:** Adjusted associations between phenols, phthalate metabolites and IQ of boys at 5 years (*N* = 452)

	Verbal IQ				Performance IQ			
	β	95% CI	*P*-value	Corrected *P*-value^c^	β	95% CI	*P*-value	Corrected *P*-value^c^
Phenols								
Parabens^a^	−0.031	[− 0.138; 0.076]	0.57	0.81	0.037	[−0.096; 0.170]	0.58	0.81
2,5 Dichlorophenol	0.015	[− 0.073; 0.102]	0.74	0.88	0.007	[−0.101; 0.115]	0.90	0.96
Benzophenone-3	0.066	[−0.025; 0.156]	0.16	0.71	0.006	[−0.105; 0.118]	0.91	0.96
Bisphenol A	−0.045	[−0.138; 0.047]	0.33	0.71	−0.059	[−0.173; 0.054]	0.31	0.71
Triclosan	0.078	[−0.012; 0.168]	0.09	0.71	0.0001	[−0.112; 0.112]	1.00	1.00
Phthalates								
MEP	−0.055	[−0.153; 0.042]	0.27	0.71	−0.023	[−0.144; 0.097]	0.70	0.88
MBP	−0.094	[−0.229; 0.041]	0.17	0.71	−0.078	[−0.243; 0.088]	0.36	0.71
MIBP	0.045	[−0.057; 0.147]	0.39	0.71	0.042	[−0.084; 0.168]	0.51	0.81
MCPP	0.136	[0.006; 0.266]	0.04	0.71	0.094	[−0.036; 0.254]	0.25	0.71
MBZP	0.005	[−0.098; 0.108]	0.92	0.96	0.089	[−0.037; 0.216]	0.17	0.71
MCOP	−0.076	[−0.178; 0.026]	0.15	0.71	−0.055	[−0.181; 0.070]	0.39	0.71
MCNP	0.019	[−0.083; 0.121]	0.72	0.88	0.060	[−0.066; 0.186]	0.35	0.71
DEHP^b^	−0.042	[−0.143; 0.059]	0.41	0.71	−0.034	[−0.158; 0.090]	0.59	0.81

Root Mean Square Error of Approximation (RMSEA): 0.048 (95% Confidence Interval (CI): 0.043; 0.053), Comparative Fit Index (CFI) = 0.930, Tucker-Lewis fit index (TLI) = 0.921

*Abbreviations*: *CI* confidence interval, *MBP* mono-n-butyl phthalate, *MBZP* monobenzyl phthalate, *MCNP* monocarboxy-isononyl phthalate, *MCOP* monocarboxy-isooctyl phthalate, *MCPP* mono(3-carboxypropyl) phthalate, *MEP* monoethyl phthalate, *MIBP* mono-isobutyl phthalate

^a^Latent variable of methyl, ethyl, propyl and butyl parabens

^b^Latent variable of di(2-ethylhexyl) phthalate metabolites: Mono(2-ethyl-5-carboxypentyl) phthalate, Mono(2-ethyl-5-hydroxyhexyl) phthalate, Mono(2-ethyl-5-oxohexyl) phthalate and Mono(2-ethylhexyl) phthalate

^c^*P*-values corrected for multiple comparisons using the Benjamini and Hochberg false discovery rate method. β = change in IQ SD associated with 1-SD increase in ln-transformed concentrations

In the sensitivity analyses, running a SEM model with only one construct for IQ led to similar results and the only significant (uncorrected *p*-value below 0.05) association observed was a positive association between MCPP and Full Scale IQ (Additional file 1: Table S4).

When we restricted our analysis to mother-child pairs with no missing values on the covariates (*N* = 419), the positive association observed between MCPP and verbal IQ was slightly attenuated (Table 4): β was 0.109 (95% CI: −0.024; 0.243) compared to 0.136 (95% CI: 0.006; 0.266) in our main analysis and a positive association between triclosan and verbal IQ emerged (*β* = 0.101, 95% CI: 0.008; 0.194 *p*-value = 0.034).

**Table 4:** Adjusted associations between phenols, phthalate metabolites and IQ of boys at 5 years in the EDEN cohort (*N* = 419 mother-son pairs, complete case analysis

	Verbal IQ				Performance IQ			
	β	95% CI	*P*value	Corrected *P*value^c^	β	95% CI	*P*value	Corrected *P*value^c^
Phenols								
Parabens^a^	−0.060	[− 0.170; 0.050]	0.28	0.76	0.033	[−0.105; 0.171]	0.64	0.76
2,5 dichlorophenol	0.017	[− 0.072; 0.107]	0.70	0.79	0.027	[−0.084; 0.138]	0.64	0.76
Benzophenone-3	0.044	[−0.049; 0.138]	0.35	0.76	−0.048	[−0.164; 0.068]	0.42	0.76
Bisphenol A	−0.032	[− 0.126; 0.063]	0.51	0.76	−0.067	[−0.184; 0.050]	0.26	0.76
Triclosan	0.101	[0.008; 0.194]	0.03	0.76	0.007	[−0.111; 0.125]	0.91	0.91
Phthalates								
MEP	−0.039	[−0.140; 0.062]	0.45	0.76	0.012	[−0.113; 0.137]	0.85	0.88
MBP	−0.103	[−0.244; 0.037]	0.15	0.76	−0.096	[−0.271; 0.078]	0.28	0.76
MIBP	0.046	[−0.060; 0.152]	0.40	0.76	0.035	[−0.096; 0.166]	0.60	0.76
MCPP	0.109	[−0.024; 0.243]	0.11	0.76	0.058	[−0.107; 0.223]	0.49	0.76
MBZP	0.028	[−0.078; 0.133]	0.61	0.76	0.112	[−0.018; 0.242]	0.09	0.76
MCOP	−0.062	[−0.170; 0.045]	0.26	0.76	−0.022	[−0.155; 0.111]	0.74	0.81
MCNP	0.027	[−0.077; 0.131]	0.61	0.76	0.062	[−0.068; 0.191]	0.35	0.76
DEHP^b^	−0.070	[−0.174; 0.033]	0.18	0.76	−0.062	[−0.191; 0.066]	0.34	0.76

Model fit indices; RMSEA = 0.049(95% CI = 0.043–0.054), CFI = 0.930, TFI = 0.920

*Abbreviations*: *CI* confidence interval, *MBP* mono-n-butyl phthalate, *MBZP* monobenzyl phthalate, *MCNP* monocarboxy-isononyl phthalate, *MCOP* monocarboxy-isooctyl phthalate, *MCPP* mono(3-carboxypropyl) phthalate, *MEP* monoethyl phthalate, *MIBP* mono-isobutyl phthalate

^a^Latent variable of methyl, ethyl, propyl and butyl parabens

^b^Latent variable of di(2-ethylhexyl) phthalate metabolites: Mono(2-ethyl-5-carboxypentyl) phthalate, Mono(2-ethyl-5-hydroxyhexyl) phthalate, Mono(2-ethyl-5-oxohexyl) phthalate and Mono(2-ethylhexyl) phthalate

^c^*P*-values corrected for multiple comparisons using the Benjamini and Hochberg false discovery rate method. β = change in IQ SD associated with 1-SD increase in ln-transformed concentrations

Using the manual based IQ scores and running multivariate linear regression models (one model per outcome and exposure) instead of SEM suggested no significant association between phenols, phthalate metabolites and the IQ scales (all *p*-values were above 0.13; Additional file 1: Table S5). Similarly, no significant association was observed after categorizing exposure in tertiles (*p*-value for heterogeneity > 0.07, Additional file 1: Table S6), except for triclosan (*p*-value for heterogeneity = 0.05), for which results were suggestive of a positive association with verbal IQ. Verbal IQ increased by 3,82, points (95% CI: 0.70; 6.93) and 2.31 points (95% CI: −0.79; 5.46) in the second and third triclosan tertile concentrations respectively (Additional file 1: Table S6).

In stratified analyses, we observed some heterogeneity in associations of IQ with benzophenone-3 and triclosan urinary concentation by center (Additional file 1: Table S7).

## Discussion

Results of our main analysis were suggestive of a positive association between maternal urinary concentrations of MCPP and verbal IQ of boys at 5–6 years that did not remain significant after correction for multiple comparisons. No other phenol or phthalate metabolite was associated with the boys’ IQ in our main analysis.

### Phthalates and the boys’ IQ

MCPP is a metabolite of di-n-octyl phthalate, used as a plasticizer in the production of plastics to improve flexibility. It is found in products such as carpet back coating, wire, cables, and adhesives [47]. Among the few studies that explored the associations between prenatal exposure to phthalates and the cognitive function, only three examined child IQ [22, 23, 28] and none measured MCPP in urine, limiting comparison. Two of these studies found no significant associations between child IQ at 6 years (*N* = 182) [23] or 11 years (*N* = 110) [22] and maternal phthalate metabolite urinary concentrations while they reported negative associations with childhood exposure to DEHP metabolites and MBP. In another study, Factor-Litvak et al., found that when boys and girls were studied altogether, MBP, MBZP and MIBP maternal urinary concentrations were inversely associated with the full-scale IQ at 7 years. After stratifying for sex, Factor-Litvak et al., reported an inverse association between MIBP and the full-scale IQ of boys at 7 years (*N* = 155) [28]. This study included Hispanics and African Americans women of inner city New-York which could limit comparability with our results that relied on a population that mostly included highly educated Caucasian European women. All three aforementioned studies had smaller population sizes than our study (*N* = 452 boys): 110 boys and girls [22], 182 boys and girls [23] and 155 boys [28] respectively. While we used IQ to evaluate the cognitive function of children, other assessments such as the Bailey Scales of Infant Development (BSID) could be used. Among studies relying on the BSID, two reported that DEHP metabolites and MEP were negatively associated with the mental index of boys at 6 months and 2 years [24, 26] while another reported positive associations between MCPP along with metabolites of di(n-butyl) phthalate (MBP and MBZP) and this mental index in boys at 2 years [25]. Reports of null associations also exist [48].

As observed in our main analysis, the positive association between MCPP and verbal IQ was not significant after correction for multiple comparisons and the effect direction was opposite to what was reported for other phthalate metabolites in a previous study on IQ [28]. Altogether, these findings did not provide strong support for an effect of MCPP on child IQ.

### Phenols and the boys’ IQ

In our study population, prenatal exposure to bisphenol A was not associated with boy’s IQ at 5 years. In line with our results, two epidemiological studies did not report associations between maternal urinary concentrations of bisphenol A and child IQ at 5 and 8 years [19, 20]. Lin et al., reported decreased Full Scale and verbal IQ scores at 7 years in association with higher levels of bisphenol A in cord blood [21]. Frequency of detection of bisphenol A in cord blood was low (56%) compared to levels detected in maternal urine (frequency of detection ranged from 86 to 99% in our study and in two previous studies by Stacy et al. and Braun et al. [19, 20]). Two other studies performed among younger children (1, 3 and 4 years versus 5–6 years in our study) and relying on the BSID, the McCarthy Scales of Children’s Abilities (MSCA) or the Behavior Rating Inventory of Executive Function-Preschool (BRIEF-P) to assess cognitive function did not report any association with prenatal exposure to bisphenol A [17] among boys but among girls, who had lower scores on the emotional control and inhibition scale of the BRIEF-P with increased prenatal exposure to bisphenol A [17].

None of the other phenols assessed was associated with IQ of boys in our main analysis, while after stratification for recruitment centers, benzophenone-3 and triclosan were associated with some IQ scores, the direction of the association varying according to IQ scale (performance versus verbal IQ) and recruitment centers. In humans, we did not identify any prior studies assessing the associations of prenatal exposure to dichlorophenol, benzophenone-3, triclosan and parabens with child cognition. In the animal literature, one rodent study attempted to assess the effects of butyl paraben on spatial memory of rats. This study reported that exposure of expectant rats to high levels of butyl paraben (200 mg/kg/day) negatively affected spatial memory in offspring [49]. Although Ali et al. suggest deleterious effects on spatial memory of rats, we did not observe such effects with the IQ of boys at 5–6 years.

#### Strengths and limitations

Strengths of our study included the larger sample size (*N* = 452 boys) compared to previous studies that examined prenatal exposure to bisphenol A and phthalates and child cognitive development among (N ranged between 64 to 266 boys [17, 19, 20, 22, 24, 26, 28]). We relied on SEM to study the association with cognitive function. SEM permitted grouping of compound esters (parabens), metabolites from similar parent compounds (DEHP metabolites) and IQ outcomes through latent variables. Use of latent variables limited the number of associations tested compared to the classical approaches that relied on the use of one regression model per outcome and exposure. Furthermore, we used the Benjamini and Hochberg FDR method to control for multiple testing and limit chance finding. One limitation of the FDR approach is that it does not take into account correlation across exposures and outcomes variables. Also, the FDR correction approach ignores that the tested compounds are not a random sample of all existing chemicals, but have, for most of them, some a priori plausibility for an effect on neurodevelopment based on the toxicological or epidemiological literature.

As most of the previous studies, we used one single urine measurement to assess exposure. For compounds with a short half-life such as phenols and phthalates, this is known to lead to exposure misclassification. The resulting measurement error is likely to be of classical type and to lead to attenuation bias in the effect estimate and to power loss, which may explain our null findings. In addition, non-classical error may arise in case we did not target the relevant exposure window. We only assessed exposure for boys. Although this is not a source of bias, it impedes drawing conclusions for female offspring. A previous study in girls has reported deleterious effect of bisphenol A on the executive function [17]. We only considered in-utero exposure, while previous studies that assessed both pre and post-natal exposure have suggested adverse IQ associations with phthalate and bisphenol A exposures during childhood and early adolescence [19, 22, 23]. We adjusted for many potential confounders, but residual confounding cannot be ruled out, for example; we did not adjust for nutritional habits and maternal IQ, which could affect either the exposure and/or IQ of the children. We only assessed exposure to phenols and phthalates, while other chemicals might affect child neurodevelopment [50]. A previous study that assessed exposure to 81 environmental factors among Spanish women did not report strong correlation for bisphenol A with any of the remaining 80 exposures, while phthalates were negatively correlated with some metals such as cadmium and copper (rho ranged between − 0.3 and − 0.5) [51], suggesting that associations of bisphenol A and phthalate with health outcomes are unlikely to be confounded by co-exposures.

## Conclusion

While we relied on a larger sample size (*N* = 452 boys) than in previous studies, our study did not provide evidence of an association between any of the phenol and phthalate biomarkers assessed in maternal urine and boy’s IQ at 5–6 years. The use of a spot sample to assess exposure may have limited our statistical power. Since phenols and phthalates may have sex-specific effects, our null findings among boys cannot be generalized to girls. Future studies should consider use of multiple urine samples per individual to assess exposure during pre and post-natal life among boys and girls.

## Additional file

Additional file 1: **Figure S1.** Path diagram illustrating associations between phenols, phthalate metabolites and FSIQ of boys in the EDEN Cohort. **Table S1.** Estimated standardized factor loadings of outcome and exposure indicator variables. **Table S2.** Associations between the covariates included in our final SEM model and boys’ IQ. **Table S3.** Spearman correlation coefficients between biomarker concentrations measured in maternal urine (*n* = 452^a^). **Table S4.** Adjusted associations between phenols, phthalate metabolites and Full Scale IQ of boys at 5 years using SEM (*N* = 452). **Table S5.** Adjusted associations between phenols, phthalate metabolites and IQ (Manual based scales) of boys at 5 years in the EDEN cohort, using multiple linear regression (*N* = 452). **Table S6.** Adjusted associations between phenols, phthalate metabolites concentrations categorized in tertiles and IQ (Manual based scales) of boys at 5 years in the EDEN cohort, using multiple linear regression (*N* = 452). **Table S7.** Adjusted associations between phenols, phthalate metabolites and IQ of boys at 5 years in the EDEN cohort, stratified for center of recruitment. (DOCX 184 kb)

## Abbreviations

CESD: Center for Epidemiologic Studies Depression Scale
CI: Confidence interval
DEHP: Di(2-ethylhexyl) phthalate
FDR: False discovery rate
IQ: Intelligence quotient
LOD: Limit of detection
MBP: Mono-n-butyl phthalate
MBZP: Monobenzyl phthalate
MCNP: Monocarboxy-isononyl phthalate
MCOP: Monocarboxy-isooctyl phthalate
MCPP: Mono(3-carboxypropyl) phthalate
MECPP: Mono(2-ethyl-5-carboxypentyl) phthalate
MEHHP: Mono(2-ethyl-5-hydroxyhexyl) phthalate
MEHP: Mono(2-ethylhexyl) phthalate
MEOHP: Mono(2-ethyl-5-oxohexyl) phthalate
MEP: Monoethyl phthalate
MIBP: Mono-isobutyl phthalate
RMSEA: Root Mean Square Error of Approximation
SD: Standard deviation
SEM: Structural Equation Model
STAI: The State-Trait Anxiety Inventory
WPPSI: Wechsler Preschool and Primary Scale of Intelligence
