# Supplementary material for: In-utero exposure to phenols and phthalates and the intelligence quotient of boys at 5 years
Source: Environ Health. 2018 Feb 20;17:17. doi: 10.1186/s12940-018-0359-0 (PMC5819230; doi:10.1186/s12940-018-0359-0)
Supplement: Supplementary file 1 — Figure S1. Path diagram illustrating associations between phenols, phthalate metabolites and FSIQ of boys in the EDEN Cohort. Table S1. Estimated standardized factor loadings of outcome and exposure indicator variables. Table S2. Associations between the covariates included in our final SEM model and boys’ IQ. Table S3. Spearman correlation coefficients between biomarker concentrations measured in maternal urine (n = 452a). Table S4. Adjusted associations between phenols, phthalate metabolites and Full Scale IQ of boys at 5 years using SEM (N = 452). Table S5. Adjusted associations between phenols, phthalate metabolites and IQ (Manual based scales) of boys at 5 years in the EDEN cohort, using multiple linear regression (N = 452). Table S6. Adjusted associations between phenols, phthalate metabolites concentrations categorized in tertiles and IQ (Manual based scales) of boys at 5 years in the EDEN cohort, using multiple linear regression (N = 452). Table S7. Adjusted associations between phenols, phthalate metabolites and IQ of boys at 5 years in the EDEN cohort, stratified for center of recruitment. (DOCX 184 kb) [file 12940_2018_359_MOESM1_ESM.docx]

Additional file 1

**In-utero exposure to phenols and phthalates and the Intelligence quotient (IQ) of boys at 5-6 years**

**List of co-authors:**

Dorothy Nakiwala^1^, Hugo Peyre^2,3^, Barbara Heude^4,5^, Jonathan Y. Bernard^4,5,6^, Rémi Béranger^7^, Rémy Slama^1^, Claire Philippat^1^, and *the EDEN mother-child study group*

**Contents**

**List of figures**

[Figure S1: Path diagram illustrating associations between phenols, phthalate metabolites and FSIQ of boys in the EDEN Cohort. 2](#_Toc500785747)

**List of tables**

[Table S1: Estimated standardized factor loadings of outcome and exposure indicator variables 3](#_Toc501626656)

[Table S2: Associations between the covariates included in our final model and boys’ IQ 4](#_Toc501626657)

[Table S3: Spearman correlation coefficients between biomarker concentrations measured in maternal urine concentrations (n = 452^a^) 5](#_Toc501626658)

[Table S4: Adjusted associations between phenols, phthalate metabolites and Full Scale IQ of boys at 5 years using SEM (N= 452) 6](#_Toc501626659)

[Table S5: Adjusted associations between phenols, phthalate metabolites and IQ (Manual based scales) of boys at 5 years in the EDEN cohort, using multiple linear regression (N= 452) 7](#_Toc501626660)

[Table S6: Adjusted associations between phenols, phthalate metabolites (concentrations categorized in tertiles) and IQ (Manual based scales) of boys at 5 years in the EDEN cohort, using multiple linear regression (N= 452) 8](#_Toc501626661)

[Table S7: Adjusted associations between phenols, phthalate metabolites and IQ of boys at 5 years in the EDEN cohort, stratified for center of recruitment 10](#_Toc501626662)


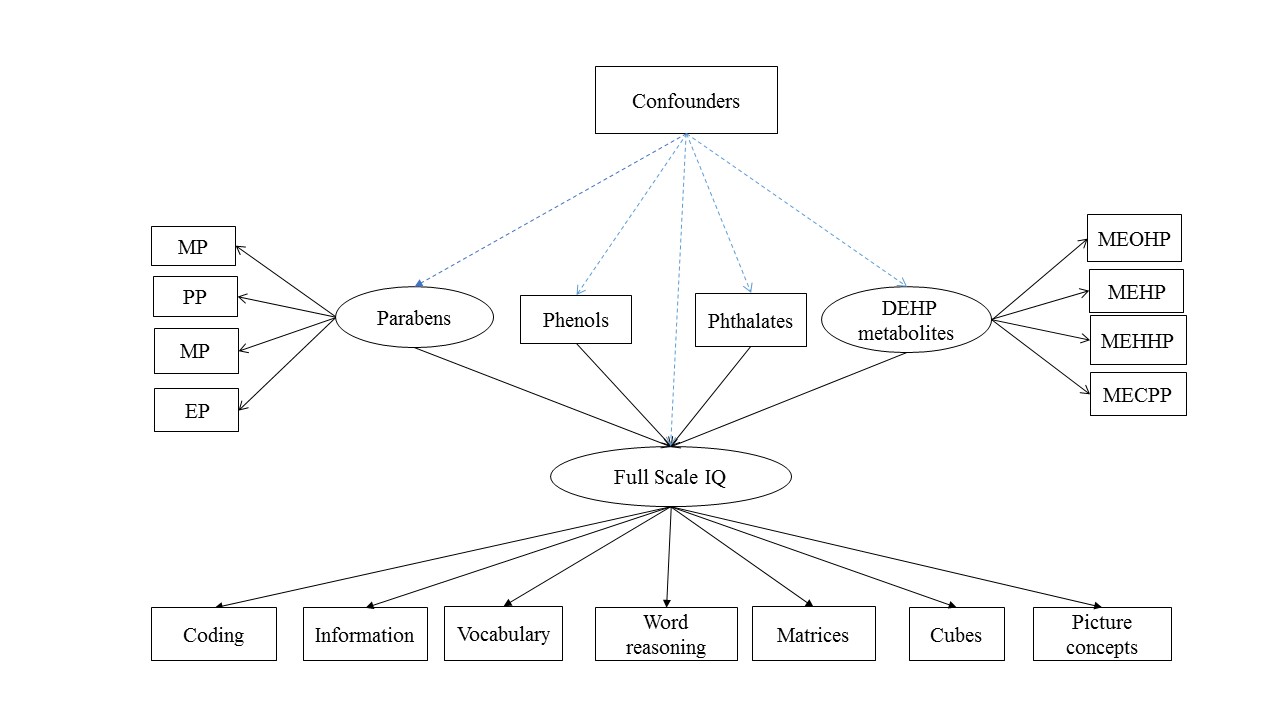


**Figure S1: Path diagram illustrating associations between phenols, phthalate metabolites and FSIQ of boys in the EDEN Cohort.** Shapes representations: circles: latent variables, rectangles: observed variables, dashed and solid arrows: linear relationships, double headed arrows: correlations. Abbreviations: MP: methyl paraben, EP: ethyl paraben, PP: propyl paraben, BP: butyl paraben, DEHP: di(2-ethylhexyl) phthalate metabolites, MECPP: mono(2-ethyl-5-carboxypentyl) phthalate, MEHHP: mono(2-ethyl-5-hydroxyhexyl) phthalate, MEOHP: mono(2-ethyl-5-oxohexyl) phthalate, MEHP: mono(2-ethylhexyl) phthalate. Phenols represents 2,5-dichlorophenol, benzophenone 3, bisphenol A and triclosan. Phthalates represents mono-n-butyl phthalate, monobenzyl phthalate, monocarboxy-isononyl phthalate, monocarboxy-isooctyl phthalate, mono(3-carboxypropyl) phthalate, monoethyl phthalate and mono-isobutyl phthalate. Confounders include maternal age, age of child at IQ assessment, HOME stimulation score, maternal body mass index before pregnancy, parental education level, parity, monthly revenue, breast feeding duration and maternal psychological difficulties during pregnancy

**Table S1: Estimated standardized factor loadings of outcome and exposure indicator variables**

| **Latent variables** | **Predictors** | **Factor loading** | **S.E** | **P-Value** | **Variance explained by latent variable** |
| --- | --- | --- | --- | --- | --- |
| Verbal IQ | Information | 0.866 | 0.017 | <0.001 | 0.749 |
|  | Vocabulary | 0.794 | 0.022 | <0.001 | 0.630 |
|  | Word reasoning | 0.864 | 0.017 | <0.001 | 0.747 |
|  |  |  |  |  |  |
| Performance IQ | Cubes | 0.743 | 0.042 | <0.001 | 0.553 |
|  | Matrices | 0.562 | 0.044 | <0.001 | 0.316 |
|  | Picture concepts | 0.564 | 0.044 | <0.001 | 0.319 |
|  |  |  |  |  |  |
| Parabens | Methylparaben | 0.892 | 0.079 | <0.001 | 0.553 |
|  | Proplyparaben | 0.819 | 0.079 | <0.001 | 0.316 |
|  | Butylparaben | 0.565 | 0.063 | <0.001 | 0.319 |
|  | Ehtylparaben | 0.584 | 0.058 | <0.001 | 0.553 |
|  |  |  |  |  |  |
| DEHP | MEHP | 0.846 | 0.013 | <0.001 | 0.716 |
|  | MEOHP | 0.999 | 0.001 | <0.001 | 0.999 |
|  | MEHHP | 0.984 | 0.002 | <0.001 | 0.969 |
|  | MECPP | 0.941 | 0.006 | <0.001 | 0.885 |

Root Mean Square Error of Approximation (RMSEA): 0.048 (95% Confidence Interval (CI): 0.043; 0.053), Comparative Fit Index (CFI) = 0.930, Tucker-Lewis fit index (TLI) = 0.921

Abbreviations: SE: standard error, DEHP: di(2-ethylhexyl) phthalate metabolites, MECPP: Mono(2-ethyl-5-carboxypentyl) phthalate, MEHHP: Mono(2-ethyl-5-hydroxyhexyl) phthalate, MEOHP: Mono(2-ethyl-5-oxohexyl) phthalate, MEHP: Mono(2-ethylhexyl) phthalate

**Table S2: Associations between the covariates included in our final model and boys’ IQ (N = 452)**

|  | **VERBAL IQ** | | | | |  | **PERFOMANCE IQ** | | | |  |
| --- | --- | --- | --- | --- | --- | --- | --- | --- | --- | --- | --- |
|  | **Crude** | | | **Multivariate^a^** | |  | **Crude** | | **Multivariate** | |  |
| **Characteristics** | **β** | | **P-value** | **β** | **P-value** |  | **β** | **P-value** | **β** | **P-value** |  |
| **Recruitment center**  Poitiers  Nancy | ref  0.12 | | 0.01 | 0.10 | 0.09 |  | ref  0.06 | 0.28 | 0.02 | 0.81 |  |
| **Parity**  Nulliparous  ≥1 child | ref  -0.17 | | 0.07 | -0.13 | 0.01 |  | ref  -0.19 | 0.10 | -0.10 | 0.10 |  |
| **Household monthly revenue (euros)**  ≤ 1500  1500 - 3000  ≥ 3000 | ref  0.29  0.45 | | <0.001  <0.001 | 0.17  0.20 | 0.01  0.01 |  | 0.16  0.27 | 0.05  0.001 | 0.02  -0.01 | 0.76  0.91 |  |
| **Smoking during pregnancy**  No  Yes | ref  0.004 | | 0.9 | 0.09 | 0.05 |  | ref  -0.07 | 0.17 | -0.02 | 0.66 |  |
| **Maternal psychological difficulties**  No  Yes | | ref  -0.12 | 0.01 | -0.06 | 0.21 |  | ref  -0.13 | 0.02 | -0.07 | 0.18 | |
| **Was breastfeed**  Never  Yes | ref  0.12 | | <0.001 | 0.10 | 0.06 |  | ref  0.20 | <0.001 | 0.11 | 0.06 |  |
| **Maternal age at pregnancy** | 0.14 | | 0.004 | 0.11 | 0.03 |  | 0.06 | 0.26 | 0.05 | 0.42 |  |
| **Total HOME score at 5 years** | 0.19 | | 0.003 | 0.10 | 0.09 |  | 0.17 | 0.06 | 0.04 | 0.58 |  |
| **Maternal Body Mass Index (kg/m^2^)** | -0.14 | | 0.004 | -0.06 | 0.21 |  | -0.12 | 0.03 | -0.26 | 0.26 |  |
| **Child age at IQ assessment** | -0.02 | | 0.62 | -0.01 | 0.81 |  | 0.03 | 0.61 | 0.04 | 0.49 |  |
| **Parental education^b^** | 0.41 | | <0.001 | 0.27 | <0.001 |  | 0.34 | <0.001 | 0.27 | <0.001 |  |

^a^ Model simultaneously adjusted for all exposures and covariates mentioned in the table

^b^ Number of years spent in school (mean of maternal and paternal education duration in years)

β = change in SD of IQ scores associated with a 1-SD increase in the covariates

**Table S3:Spearman correlation coefficients between biomarker concentrations measured in maternal urine concentrations (n = 452^a^)**

|  | **DC4** | **DC5** | **BPA** | **BP3** | **TCS** | **MP** | **EP** | **PP** | **BP** | **MEP** | **MBP** | **MiBP** | **MCPP** | **MBzP** | **MEHP** | **MEOHP** | **MEHHP** | **MECPP** | **MCOP** | **MCNP** |
| --- | --- | --- | --- | --- | --- | --- | --- | --- | --- | --- | --- | --- | --- | --- | --- | --- | --- | --- | --- | --- |
| **DC4** | 1.00 |  |  |  |  |  |  |  |  |  |  |  |  |  |  |  |  |  |  |  |
| **DC5** | 0.69 | 1.00 |  |  |  |  |  |  |  |  |  |  |  |  |  |  |  |  |  |  |
| **BPA** | 0.01 | 0.02 | 1.00 |  |  |  |  |  |  |  |  |  |  |  |  |  |  |  |  |  |
| **BP3** | 0.11 | 0.06 | 0.05 | 1.00 |  |  |  |  |  |  |  |  |  |  |  |  |  |  |  |  |
| **TCS** | 0.37 | 0.03 | -0.05 | 0.14 | 1.00 |  |  |  |  |  |  |  |  |  |  |  |  |  |  |  |
| **MP** | 0.06 | 0.00 | 0.01 | 0.19 | 0.12 | 1.00 |  |  |  |  |  |  |  |  |  |  |  |  |  |  |
| **EP** | 0.14 | 0.01 | -0.03 | 0.17 | 0.14 | 0.58 | 1.00 |  |  |  |  |  |  |  |  |  |  |  |  |  |
| **PP** | 0.08 | -0.01 | -0.05 | 0.17 | 0.16 | 0.82 | 0.48 | 1.00 |  |  |  |  |  |  |  |  |  |  |  |  |
| **BP** | 0.10 | -0.02 | 0.00 | 0.23 | 0.17 | 0.55 | 0.72 | 0.46 | 1.00 |  |  |  |  |  |  |  |  |  |  |  |
| **MEP** | 0.15 | 0.06 | 0.05 | 0.18 | 0.20 | 0.30 | 0.15 | 0.31 | 0.22 | 1.00 |  |  |  |  |  |  |  |  |  |  |
| **MBP** | 0.01 | 0.08 | 0.14 | 0.12 | -0.05 | -0.01 | 0.08 | 0.03 | 0.04 | 0.10 | 1.00 |  |  |  |  |  |  |  |  |  |
| **MiBP** | -0.05 | 0.05 | 0.13 | 0.07 | -0.06 | 0.08 | 0.03 | 0.05 | 0.00 | 0.04 | 0.42 | 1.00 |  |  |  |  |  |  |  |  |
| **MCPP** | 0.06 | 0.08 | 0.25 | 0.02 | -0.06 | 0.00 | 0.03 | 0.00 | -0.02 | 0.00 | 0.61 | 0.26 | 1.00 | 0.26 |  |  |  |  |  |  |
| **MBzP** | -0.03 | -0.02 | 0.19 | 0.09 | -0.05 | 0.01 | 0.01 | 0.01 | -0.05 | 0.07 | 0.43 | 0.38 | 0.26 | 1.00 |  |  |  |  |  |  |
| **MEHP** | 0.07 | 0.10 | 0.24 | 0.03 | 0.00 | -0.03 | -0.01 | -0.03 | -0.05 | 0.07 | 0.22 | 0.25 | 0.24 | 0.27 | 1.00 |  |  |  |  |  |
| **MEOHP** | 0.09 | 0.11 | 0.24 | 0.03 | 0.02 | -0.04 | 0.00 | -0.07 | -0.08 | 0.05 | 0.31 | 0.28 | 0.32 | 0.35 | 0.84 | 1.00 |  |  |  |  |
| **MEHHP** | 0.09 | 0.12 | 0.25 | 0.02 | 0.00 | -0.04 | 0.00 | -0.08 | -0.07 | 0.05 | 0.29 | 0.27 | 0.31 | 0.33 | 0.82 | 0.98 | 1.00 |  |  |  |
| **MECPP** | 0.10 | 0.11 | 0.21 | 0.00 | -0.01 | -0.07 | -0.03 | -0.09 | -0.11 | 0.01 | 0.24 | 0.25 | 0.26 | 0.30 | 0.78 | 0.93 | 0.90 | 1.00 |  |  |
| **MCOP** | 0.06 | 0.03 | 0.24 | 0.04 | 0.01 | -0.01 | -0.02 | 0.00 | -0.02 | 0.02 | 0.12 | 0.21 | 0.26 | 0.21 | 0.30 | 0.34 | 0.34 | 0.38 | 1.00 |  |
| **MCNP** | 0.15 | 0.08 | 0.20 | 0.01 | 0.03 | 0.01 | 0.04 | 0.03 | 0.01 | -0.01 | 0.12 | 0.11 | 0.32 | 0.16 | 0.21 | 0.26 | 0.26 | 0.26 | 0.42 | 1.00 |

Abbreviations: BP: butylparaben, BPA: bisphenol A, BP3: benzophenone-3, DCP: dichlorophenol, EP: ethylparaben, MBP: mono-n-butyl phthalate, MBzP: monobenzyl phthalate, MCNP: monocarboxy-isononyl phthalate, MCOP: monocarboxy-isooctyl phthalate, MCPP: mono(3-carboxypropyl) phthalate, MECPP: mono(2-ethyl-5-carboxypentyl) phthalate, MEHHP: mono(2-ethyl-5-hydroxyhexyl) phthalate, MEHP: mono(2-ethylhexyl) phthalate, MEOHP: mono(2-ethyl-5-oxohexyl) phthalate, MEP:monoethyl phthalate, MiBP: mono-isobutyl phthalate, MP: methylparaben, PP: propylparaben, TCS: triclosan.

^a^Mother-son pairs with IQ scores and biomarker concentration assessment.

**Table S4:Adjusted associations between phenols, phthalate metabolites and Full Scale IQ of boys at 5 years using SEM (N= 452)**

|  | β | 95% CI | P-value | Corrected  P-value^c^ |
| --- | --- | --- | --- | --- |
| Phenols | | | | |
| Parabens^a^ | -0.022 | [-0.130; 0.084] | 0.68 | 0.50 |
| 2,5 Dichlorophenol | 0.018 | [-0.070; 0.104] | 0.69 | 0.74 |
| Benzophenone-3 | 0.060 | [-0.030; 0.149] | 0.19 | 0.50 |
| Bisphenol A | -0.049 | [-0.140; 0.043] | 0.30 | 0.55 |
| Triclosan | 0.070 | [-0.020; 0.159] | 0.12 | 0.50 |
| Phthalates | | | | |
| MEP | -0.055 | [-0.150; 0.044] | 0.27 | 0.55 |
| MBP | -0.093 | [-0.230; 0.038] | 0.17 | 0.50 |
| MIBP | 0.047 | [-0.053; 0.150] | 0.37 | 0.56 |
| MCPP | 0.135 | [0.009; 0.268] | 0.04 | 0.50 |
| MBZP | 0.015 | [-0.086; 0.118] | 0.77 | 0.77 |
| MCOP | -0.076 | [-0.179; 0.024] | 0.14 | 0.50 |
| MCNP | 0.025 | [-0.079; 0.124] | 0.63 | 0.74 |
| DEHP^b^ | -0.044 | [-0.144 0.056] | 0.39 | 0.55 |

Root Mean Square Error of Approximation (RMSEA): 0.05 (95% Confidence Interval (CI): 0.046; 0.055), Comparative Fit Index (CFI) = 0.912, Tucker-Lewis fit index (TLI) = 0.907. Abbreviations: CI: confidence interval, MBP: mono-n-butyl phthalate, MBZP: monobenzyl phthalate, MCNP: monocarboxy-isononyl phthalate, MCOP: monocarboxy-isooctyl phthalate, MCPP: mono(3-carboxypropyl) phthalate, MEP: monoethyl phthalate and MIBP: mono-isobutyl phthalate.

^a^Latent variable of methyl, ethyl, propyl and butyl parabens

^b^Latent variable of di(2-ethylhexyl) phthalate metabolites: Mono(2-ethyl-5-carboxypentyl) phthalate, Mono(2-ethyl-5-hydroxyhexyl) phthalate, Mono(2-ethyl-5-oxohexyl) phthalate and Mono(2-ethylhexyl) phthalate.

^c^ P-values corrected for multiple comparisons using the Benjamini and Hochberg false discovery rate method.

**Table S5:Adjusted associations between phenols, phthalate metabolites and IQ (Manual based scales) of boys at 5 years in the EDEN cohort, using multiple linear regression (N= 452)**

|  | **Full Scale IQ** | | |  | **Verbal IQ** | | |  | **Performance IQ** | | |
| --- | --- | --- | --- | --- | --- | --- | --- | --- | --- | --- | --- |
|  | **β** | **95% CI** | **P-value** |  | **β** | **95% CI** | **P-value** |  | **β** | **95% CI** | **P-value** |
| **Phenols** | | | | | | | | | | | |
| Parabens^a^ | 0.14 | [-0.65; 0.93] | 0.73 |  | -0.02 | [-0.83; 0.79] | 0.96 |  | 0.50 | [-0.33; 1.33] | 0.24 |
| Dichlorophenol^b^ | 0.45 | [-0.38; 1.27] | 0.29 |  | 0.21 | [-0.63; 1.05] | 0.62 |  | 0.20 | [-0.66; 1.06] | 0.65 |
| Benzophenone-3 | 0.01 | [-0.73; 0.76] | 0.97 |  | 0.21 | [-0.55; 0.97] | 0.59 |  | 0.01 | [-0.77; 0.80] | 0.98 |
| Bisphenol A | -0.65 | [-2.31; 1.02] | 0.45 |  | -0.88 | [-2.58; 0.82] | 0.31 |  | -0.46 | [-2.22; 1.29] | 0.60 |
| Triclosan | 0.04 | [-0.46; 0.55] | 0.87 |  | 0.32 | [-0.19; 0.84] | 0.22 |  | -0.28 | [-0.82; 0.25] | 0.30 |
| **Phthalates** | | | | | | | | | | | |
| MEP | -0.56 | [-1.77; 0.65] | 0.37 |  | -0.72 | [-1.95; 0.51] | 0.25 |  | -0.07 | [-1.33; 1.20] | 0.92 |
| MBP | -0.09 | [-1.30; 1.13] | 0.89 |  | -0.28 | [-1.51; 0.95] | 0.66 |  | -0.10 | [-1.37; 1.17] | 0.87 |
| MIBP | 0.38 | [-1.14; 1.91] | 0.62 |  | 0.15 | [-1.40; 1.71] | 0.85 |  | 0.89 | [-0.71; 2.50] | 0.27 |
| MCPP | 1.05 | [-0.55; 2.65] | 0.20 |  | 0.73 | [-0.90; 2.36] | 0.38 |  | 0.95 | [-0.73; 2.63] | 0.27 |
| MBZP | 0.14 | [-1.12; 1.40] | 0.83 |  | -0.31 | [-1.60; 0.98] | 0.64 |  | 0.76 | [-0.57; 2.08] | 0.26 |
| MCOP | -0.57 | [-2.03; 0.90] | 0.45 |  | -1.06 | [-2.55; 0.42] | 0.16 |  | -0.02 | [-1.55; 1.52] | 0.98 |
| MCNP | 0.63 | [-0.65; 1.91] | 0.33 |  | 0.10 | [-1.21; 1.41] | 0.88 |  | 1.02 | [-0.32; 2.37] | 0.13 |
| DEHP^c^ | -0.42 | [-1.92; 1.08] | 0.58 |  | -0.75 | [-2.27; 0.78] | 0.34 |  | -0.05 | [-1.63; 1.52] | 0.95 |

Abbreviations: CI: confidence interval, MBP: mono-n-butyl phthalate, MBZP: monobenzyl phthalate, MCNP: monocarboxy-isononyl phthalate, MCOP: monocarboxy-isooctyl phthalate, MCPP: mono(3-carboxypropyl) phthalate, MEP: monoethyl phthalate and MIBP: mono-isobutyl phthalate.

^a^Molar sum of methyl, ethyl, propyl and butyl parabens

^b^Molar sum of 2,4 and 2,5 Dichlorophenol

^c^Molar sum of di(2-ethylhexyl) phthalate metabolites: Mono(2-ethyl-5-carboxypentyl) phthalate, Mono(2-ethyl-5-hydroxyhexyl) phthalate, Mono(2-ethyl-5-oxohexyl) phthalate and Mono(2-ethylhexyl) phthalate.

**Table S6: Adjusted associations between phenols, phthalate metabolites (concentrations categorized in tertiles) and IQ (Manual based scales) of boys at 5 years in the EDEN cohort, using multiple linear regression (N= 452)**

|  | **Full Scale IQ** | | | |  | **Verbal IQ** | | | |  | **Performance IQ** | | | |
| --- | --- | --- | --- | --- | --- | --- | --- | --- | --- | --- | --- | --- | --- | --- |
|  | **β** | **95% CI** | **Phet** | **ptrend** |  | **β** | **95% CI** | **Phet** | **ptrend** |  | **β** | **95% CI** | **Phet** | **ptrend** |
| **phenols** | | | | | | | | | | | | | | |
| ∑Parabens |  |  |  |  |  |  |  |  |  |  |  |  |  |  |
| T2 | 2,35 | [-0.66; 5.37] | 0.31 | 0.48 |  | 1.50 | [-1.57; 4.58] | 0.27 | 0.56 |  | 2.70 | [-0.47; 5.87] | 0.10 | 0.04 |
| T3 | 0.98 | [-2.12; 4.08] |  |  |  | -1.09 | [-4.24; 2.06] |  |  |  | 3.28 | [0.04; 6.53] |  |  |
| ∑ Dichlorophenol |  |  |  |  |  |  |  |  |  |  |  |  |  |  |
| T2 | 0.32 | [-2.72; 3.36] | 0.77 | 0.47 |  | 0.53 | [-2.57; 3.63] | 0.94 | 0.90 |  | -0.14 | [-3.34; 3.06] | 0.94 | 0.77 |
| T3 | 1.06 | [-1.94; 4.06] |  |  |  | 0.22 | [-2.84; 3.29] |  |  |  | 0.42 | [-2.75; 3.58] |  |  |
| Benzophenone-3 |  |  |  |  |  |  |  |  |  |  |  |  |  |  |
| T2 | -0.23 | [-3.25; 2.80] | 0.96 | 0.87 |  | -0.24 | [-3.32; 2.84] | 0.65 | 0.43 |  | 0.33 | [-2.86; 3.51] | 0.81 | 0.51 |
| T3 | 0.19 | [-2.89; 3.28] |  |  |  | 1.12 | [-2.02; 4.26] |  |  |  | 1.05 | [-2.19; 4.30] |  |  |
| Bisphenol A |  |  |  |  |  |  |  |  |  |  |  |  |  |  |
| T2 | 0.32 | [-2.80; 3.44] | 0.95 | 0.89 |  | -0.36 | [-3.52; 2.81] | 0.48 | 0.25 |  | 0.56 | [-2.70; 3.83] | 0.83 | 0.55 |
| T3 | -0.20 | [-3.23; 2.83] |  |  |  | -1.80 | [-4.89; 1.28] |  |  |  | 0.97 | [-2.22; 4.16] |  |  |
| Triclosan |  |  |  |  |  |  |  |  |  |  |  |  |  |  |
| T2 | 3.02 | [-0.03; 6.07] | 0.13 | 0.59 |  | 3.82 | [0.70; 6.93] | 0.05 | 0.14 |  | 1.90 | [-1.31; 5.11] | 0.18 | 0.53 |
| T3 | 0.81 | [-2.24; 3.86] |  |  |  | 2.31 | [-0.79; 5.40] |  |  |  | -1.04 | [-4.25; 2.16] |  |  |
| **Phthalates** |  |  |  |  |  |  |  |  |  |  |  |  |  |  |
| MEP |  |  |  |  |  |  |  |  |  |  |  |  |  |  |
| T2 | -1.89 | [-4.88; 1.10] | 0.46 | 0.50 |  | -1.71 | [-4.75; 1.33] | 0.42 | 0.42 |  | -1.85 | [-4.99; 1.30] | 0.30 | 0.30 |
| T3 | -1.09 | [-4.18; 2.00] |  |  |  | -1.89 | [-5.03; 1.26] |  |  |  | 0.49 | [-2.75; 3.74] |  |  |
| MBP |  |  |  |  |  |  |  |  |  |  |  |  |  |  |
| T2 | 1.35 | [-1.67; 4.38] | 0.67 | 0.81 |  | 1.38 | [-1.70; 4.46] | 0.44 | 0.24 |  | 1.32 | [-1.87; 4.50] | 0.63 | 0.76 |
| T3 | 0.50 | [-2.62; 3.62] |  |  |  | -0.52 | [-3.69; 2.65] |  |  |  | 1.43 | [-1.85; 4.71] |  |  |
| MIBP |  |  |  |  |  |  |  |  |  |  |  |  |  |  |
| T2 | -1.50 | [-4.47; 1.47] | 0.13 | 0.27 |  | -0.51 | [-3.56; 2.54] | 0.82 | 0.66 |  | -2.45 | [-5.55; 0.65] | 0.12 | 0.41 |
| T3 | 1.72 | [-1.35; 4.78] |  |  |  | 0.52 | [-2.62; 3.65] |  |  |  | 2.76 | [-0.44; 5.96] |  |  |
| MCPP |  |  |  |  |  |  |  |  |  |  |  |  |  |  |
| T2 | 2.83 | [-0.14; 5.80] | 0.15 | 0.20 |  | 3.07 | [0.04; 6.10] | 0.14 | 0.74 |  | 2.53 | [-0.60; 5.66] | 0.21 | 0.09 |
| T3 | 2.18 | [-0.85; 5.21] |  |  |  | 1.67 | [-1.42; 4.75] |  |  |  | 2.37 | [-0.82; 5.56] |  |  |
| MBZP |  |  |  |  |  |  |  |  |  |  |  |  |  |  |
| T2 | 0.19 | [-2.85; 3.23] | 0.98 | 0.85 |  | -2.03 | [-5.13; 1.06] | 0.40 | 0.38 |  | 2.20 | [-0.99; 5.38] | 0.32 | 0.17 |
| T3 | 0.31 | [-2.75; 3.36] |  |  |  | -1.56 | [-4.67; 1.55] |  |  |  | 2.11 | [-1.10; 5.32] |  |  |
| MCOP |  |  |  |  |  |  |  |  |  |  |  |  |  |  |
| T2 | -3.53 | [-6.55; -0.51] | 0.07 | 0.19 |  | -2.57 | [-5.66; 0.52] | 0.15 | 0.35 |  | -2.92 | [-6.11; 0.27] | 0.17 | 0.22 |
| T3 | -2.06 | [-5.11; 0.98] |  |  |  | -2.80 | [-5.89; 0.30] |  |  |  | -0.71 | [-3.91; 2.49] |  |  |
| MCNP |  |  |  |  |  |  |  |  |  |  |  |  |  |  |
| T2 | -0.80 | [-3.83; 2.23] | 0.69 | 0.66 |  | -0.11 | [-3.21; 2.98] | 1.00 | 0.08 |  | -1.74 | [-4.92; 0.27] | 0.26 | 0.67 |
| T3 | 0.54 | [-2.46; 3.54] |  |  |  | -0.14 | [-3.19; 2.92] | |  |  | 0.94 | [-2.20; 4.09] |  |  |
| ∑DEHP |  |  |  |  |  |  |  |  |  |  |  |  |  |  |
| T2 | -0.68 | [-3.73; 2.37] | 0.82 | 0.84 |  | 0.37 | [-2.72; 3.47] | 0.96 | 0.93 |  | -0.40 | [-3.59; 2.80] | 0.81 | 0.45 |
| T3 | 0.26 | [-2.80; 3.33] |  |  |  | -0.01 | [-3.12; 3.13] |  |  |  | 0.65 | [-2.58; 3.88] |  |  |

Abbreviations: CI: confidence interval, T2: tertile 2, T3: tertile 3, MBP: mono-n-butyl phthalate, MBZP: monobenzyl phthalate, MCNP: monocarboxy-isononyl phthalate, MCOP: monocarboxy-isooctyl phthalate, MCPP: mono(3-carboxypropyl) phthalate, MEP: monoethyl phthalate and MIBP: mono-isobutyl phthalate.

^a^Latent variable of methyl, ethyl, propyl and butyl parabens

^b^Latent variable of di(2-ethylhexyl) phthalate metabolites: Mono(2-ethyl-5-carboxypentyl) phthalate, Mono(2-ethyl-5-hydroxyhexyl) phthalate, Mono(2-ethyl-5-oxohexyl) phthalate and Mono(2-ethylhexyl) phthalate.

**Table S7: Adjusted associations between phenols, phthalate metabolites and IQ of boys at 5 years in the EDEN cohort, stratified for center of recruitment**

|  | **POITIERS (N= 285)** | | | | | | | | |  | **NANCY (N=168)** | | | | | | | | |
| --- | --- | --- | --- | --- | --- | --- | --- | --- | --- | --- | --- | --- | --- | --- | --- | --- | --- | --- | --- |
|  | **VERBAL IQ** | | | |  | **PERFORMANCE IQ** | | | |  | **VERBAL IQ** | | | |  | **PERFORMANCE IQ** | | | |
|  | **β** | **95% CI** | **P-value** | **Corrected P-valuec** |  | **β** | **95% CI** | **P-value** | **Corrected P-valuec** |  | **β** | **95% CI** | **P-value** | **Corrected P-valuec** |  | **β** | **95% CI** | **P-value** | **Corrected P-valuec** |
|  |  |  |  |  |  |  |  |  |  |  |  |  |  |  |  |  |  |  |  |
| **Phenols** | | | |  |  |  |  |  |  |  |  |  |  |  |  |  |  |  |  |
| PBM^a^ | -0.07 | [-0.199; 0.061] | 0.3 | 0.78 |  | -0.04 | [-0.195; 0.126] | 0.67 | 0.95 |  | -0.02 | [-0.187; 0.150] | 0.83 | 0.94 |  | 0.08 | [-0.136; 0.288] | 0.48 | 0.94 |
| DC5 | 0.01 | [-0.097; 0.120] | 0.84 | 0.95 |  | 0 | [-0.133; 0.130] | 0.99 | 0.99 |  | 0.02 | [-0.134; 0.164] | 0.84 | 0.94 |  | -0.01 | [-0.180; 0.154] | 0.88 | 0.94 |
| BP3 | 0.12 | [0.012; 0.228] | 0.03 | 0.48 |  | 0.12 | [-0.011; 0.252] | 0.07 | 0.62 |  | -0.01 | [-0.172; 0.152] | 0.9 | 0.94 |  | -0.26 | [-0.433; -0.077] | 0.01 | 0.07 |
| BPA | -0.01 | [-0.123; 0.101] | 0.84 | 0.95 |  | -0.05 | [-0.189; 0.084] | 0.45 | 0.95 |  | -0.09 | [-0.238; 0.063] | 0.25 | 0.58 |  | -0.02 | [-0.190; 0.148] | 0.81 | 0.94 |
| TCS | -0.01 | [-0.121; 0.105] | 0.89 | 0.95 |  | -0.11 | [-0.246; 0.028] | 0.12 | 0.66 |  | 0.22 | [0.067; 0.363] | <0.001 | 0.07 |  | 0.18 | [0.008; 0.344] | 0.04 | 0.35 |
| **Phthalates** | | |  |  |  |  |  |  |  |  |  |  |  |  |  |  |  |  |  |
| MEP | 0.02 | [-0.108; 0.139] | 0.81 | 0.95 |  | 0.04 | [-0.109; 0.190] | 0.6 | 0.95 |  | -0.09 | [-0.248; 0.069] | 0.27 | 0.58 |  | -0.03 | [-0.209; 0.150] | 0.75 | 0.94 |
| MBP | -0.17 | [-0.332; -0.011] | 0.04 | 0.48 |  | -0.13 | [-0.323; 0.067] | 0.2 | 0.73 |  | -0.01 | [-0.246; 0.232] | 0.96 | 0.96 |  | -0.07 | [-0.343; 0.198] | 0.6 | 0.94 |
| MIBP | 0.02 | [-0.105; 0.148] | 0.74 | 0.95 |  | 0.04 | [-0.118; 0.189] | 0.65 | 0.95 |  | 0.15 | [-0.016; 0.314] | 0.08 | 0.49 |  | 0.05 | [-0.134; 0.237] | 0.59 | 0.94 |
| MCPP | 0.11 | [-0.043; 0.271] | 0.16 | 0.68 |  | 0.08 | [-0.108; 0.272] | 0.4 | 0.94 |  | 0.14 | [-0.091; 0.367] | 0.24 | 0.58 |  | 0.05 | [-0.213; 0.303] | 0.73 | 0.94 |
| MBZP | -0.01 | [-0.137; 0.123] | 0.92 | 0.95 |  | 0.09 | [-0.071; 0.244] | 0.28 | 0.78 |  | -0.02 | [-0.193; 0.150] | 0.81 | 0.94 |  | 0.11 | [-0.082; 0.301] | 0.26 | 0.58 |
| MCOP | 0.01 | [-0.120; 0.140] | 0.88 | 0.95 |  | -0.12 | [-0.279; 0.034] | 0.13 | 0.66 |  | -0.12 | [-0.296; 0.048] | 0.16 | 0.58 |  | 0.15 | [-0.041; 0.340] | 0.13 | 0.54 |
| MCNP | -0.01 | [-0.146; 0.126] | 0.88 | 0.95 |  | 0.09 | [-0.071; 0.259] | 0.27 | 0.78 |  | 0.02 | [-0.133; 0.165] | 0.84 | 0.94 |  | 0.03 | [-0.141; 0.193] | 0.76 | 0.94 |
| DEHP^b^ | 0.02 | [-0.115; 0.150] | 0.8 | 0.95 |  | 0.03 | [-0.134; 0.190] | 0.74 | 0.95 |  | -0.13 | [-0.282; 0.027] | 0.11 | 0.54 |  | -0.1 | [-0.277; 0.073] | 0.25 | 0.58 |

Model fit indices for Poitiers; RMSEA=0.046(95% CI= 0.043-0.054), CFI=0.937, TFI=0.929

Model fit indices for Nancy; RMSEA=0.047(95% CI= 0.037-0.057), CFI=0.935, TFI=0.926

Abbreviations; DC5: 2,5 dichlorophenol, BP3: benzophenone-3, BPA: bisphenol A, TCS: triclosan, MEP: monoethyl phthalate, MBP: mono-n-butyl phthalate, MIBP: mono-isobutyl phthalate, MCPP: mono(3-carboxypropyl) phthalate, MBZP: monobenzyl phthalate, MCOP: monocarboxy-isooctyl phthalate, MCNP: monocarboxy-isononyl phthalate

^a^Latent variable of methyl, ethyl, propyl and butyl parabens

^b^Latent variable of di(2-ethylhexyl) phthalate metabolites: Mono(2-ethyl-5-carboxypentyl) phthalate, Mono(2-ethyl-5-hydroxyhexyl) phthalate, Mono(2-ethyl-5-oxohexyl) phthalate and Mono(2-ethylhexyl) phthalate.
